# Molecular and Clinical Characterization of *Giardia duodenalis* Infection in Preschool Children from Lisbon, Portugal

**DOI:** 10.1155/2013/252971

**Published:** 2013-09-08

**Authors:** Filipa Santana Ferreira, Rita Alexandre dos Santos Soares de Bellegarde Machado Sá da Bandeira, Cláudia Alexandra Cecílio de Sampaio Ferreira Constantino, Ana Maria Teixeira Duarte Cancela da Fonseca, Joana da Graça Matias Gomes, Rúben Miguel Lopes Rodrigues, Jorge Luís Marques da Silva Atouguia, Sónia Chavarria Alves Ferreira Centeno-Lima

**Affiliations:** ^1^Unidade de Clínica Tropical e Centro de Malária e Doenças Tropicais-LA, Instituto de Higiene e Medicina Tropical, Universidade Nova de Lisboa, Rua da Junqueira 100, 1349-008 Lisboa, Portugal; ^2^Departamento de Pediatria Médica, Hospital Dona Estefânia, Rua Jacinta Marto, 1169-045 Lisboa, Portugal; ^3^Serviço de Pediatria Instituto Português de Oncologia de Lisboa Francisco Gentil, Rua Professor Lima Bastos, 1099-023 Lisboa, Portugal; ^4^Graduated Program in Areas of Basic and Applied Biology, Instituto de Ciências Biomédicas Abel Salazar, University of Porto, Rua Dr. Roberto Frias, s/n, 4200-465 Porto, Portugal

## Abstract

*Giardia duodenalis* is the most prevalent intestinal protozoan infection especially in children. In Portugal scarce data are available relative to this infection in preschoolers. The present study was conducted from April to July 2009 in public preschools in Lisbon enrolling 316 children. Stool examination was performed through microscopy. Molecular analysis was conducted in all positive samples for *G. duodenalis* in order to determine the assemblage and subassemblage of this parasite. Eight of the preschoolers studied children (2.5%, 8/316) were infected with *G. duodenalis*. Additionally the brother of one of the infected children was also infected. Genotyping analysis targeting *ssu-rRNA* and **β**-*giardin* loci revealed six infections with assemblage A and 3 with assemblage B. Sub-assemblage determination was possible in four of the samples, with three A2 and one A3. The limited number of cases precluded an association of a determined symptom with an assemblage. The data presented here show the relevance of considering *G. duodenalis* analysis in children with intestinal complaints even in developed countries.

## 1. Introduction

Giardiasis is a widespread intestinal disease caused by *Giardia duodenalis*. This protozoan parasite has a global distribution, infecting humans and a wide range of mammalian hosts [1]. The prevalence of giardiasis in humans in developed countries is 2–7% [2], while it may vary between 20 and 30% in developing countries [3].

The spectrum of clinical manifestations in human giardiasis is relatively variable, ranging from the absence of symptoms to acute or chronic diarrhea, dehydration, abdominal pain, nausea, vomiting, and weight loss [4]. Children are especially affected, with more severe consequences than adults. However, the impact of *Giardia* infection in children development is not clear. Some studies showed detrimental effects on nutritional status and poorer cognitive function on children with giardiasis [5–8], while others showed that giardiasis did not affect childhood growth [9]. Host factors, such as immune status, nutritional status, and age, are recognized as important determinants for the severity of infection [10]. However, studies on the possible association between *G. duodenalis *assemblages and the severity of the disease have proved thus far to be inconsistent [11].

Molecular tools have demonstrated that *G. duodenalis* is a species complex comprising at least eight assemblages (A–H), among which only A and B were found infecting humans [12].

Previous studies focused on the prevalence of giardiasis in preschool children (3-4%) [13, 14], on *G. duodenalis* assemblages determination in humans [13, 15], animals, and water [15, 16], and, more recently, on prevalence and risk factors for *G. duodenalis* infection among children [17].

The aim of this study was to determine whether there was any *G. duodenalis* in preschool children enrolled, the assemblages and their relation to clinical data from the city of Lisbon.

## 2. Material and Methods

### 2.1. Study Design, Population, and Sample

A cross-sectional study was conducted from April to July 2009. The population in study were all preschoolers (3306 children) attending that year the public preschools under the supervision of the Lisbon City Hall. The selection of the kindergartens where the study was conducted was decided by the Lisbon City aiming at reflecting a wider socioeconomic of the families and geographic dispersion of the children. The number of children involved was of 685.

A total of 316 (46.1%, 316/685) preschool children, aged 3–6, attending the selected kindergartens of the network of public schools, were enrolled in this study. All children in the schools were invited to participate. The enrolled children were those whose parents collected the stool samples and signed the informed consent. 

### 2.2. Sample Collection

A meeting was held with the director of each school as well as with the parents to explain the study objectives, prior to sample collection. 

The stool containers were delivered to the parents or guardians on a Friday. The parents/guardians were told to collect three stool samples in consecutive days without any pharmacologic induction and stored at 4°C till Monday morning, when the team went to the schools to collect all the samples.

### 2.3. Microscopy

The fresh stool samples were screened for *G. duodenalis *and other intestinal parasites through microscopic analysis in saline and also in iodine. Furthermore, the formolether concentration method was also performed to increase the sensitivity of the detection. All samples were screened by three different microscopists, for cross-check results. Positive samples for *G. duodenalis *were kept in filter paper (Generation Card Kit, Qiagen) and preserved at −20°C for further analysis.

### 2.4. DNA Extraction and PCR Amplification

DNA was extracted from samples preserved in filter paper. A DNA extraction protocol for dried blood spots (Generation Capture Card Kit, Qiagen) was adapted for stool samples. Changes to the original protocol included tripling the solution volumes used, except for the final elution step (100 *μ*L) [18]. For some samples, DNA was reextracted using elution volumes of 25 *μ*L. All samples were amplified using primers targeting the small subunit ribosomal RNA (*ssu-rRNA*) [19, 20] and **β*-giardin* (*bg*) loci [21, 22].

Amplification reactions were performed using 2 *μ*L of DNA template in a final volume of 25 *μ*L, using illustra PuReTaq Ready-To-Go PCR beads (GE Healthcare, UK). Both positive (DNA isolated from the Portland-1 strain (ATCC 30888DLGC Promochem) and negative controls (no template added) were included in each series of PCR reactions. PCR products were visualized on 2% agarose gel stained with ethidium bromide.

### 2.5. Sequence Analysis

For sequence analysis, PCR products were purified using JETQUICK Gel Extraction Spin Kit/50 (Genomed, Germany) according to the manufacturer's instructions. DNA sequencing reactions were carried out in both directions using primers GiarF/GiarR for *ssu-rRNA* gene fragment (175 bp) [20] and those described previously [22] for **β*-giardin* gene fragment (511 bp). Sequences obtained in this study were aligned with previously published sequences of *G. duodenalis* isolates available in the GenBank database, using ClustalW.

### 2.6. Clinical Data and Treatment

A questionnaire for clinical data relative to the children was filled by each participant's parent or guardian.

All children infected with *G. duodenalis* were assisted by a paediatric doctor from the Tropical Diseases Clinic of the Institute of Hygiene and Tropical Medicine and treated for the infection with 15 mg/kg/day of metronidazole divided in three daily doses, for seven days. Three weeks after the treatment new stool samples were obtained in order to confirm the treatment efficacy. If a child was infected with any intestinal parasite, the remaining members of the household were invited to collect their stools that were also screened for intestinal parasites. 

### 2.7. Ethical Considerations

The present study was submitted and approved by the Ethical Committee of the Institute of Hygiene and Tropical Medicine, Lisbon. Written informed consent was obtained from all parents or the legal guardians of the children participating in the study. 

## 3. Results

### 3.1. Parasitological Results

From the 316 children participating in this study, 166 (52.5%) were males and 150 (47.5%) were females. The mean age was 5.03, ranging between three and six years old. *G. duodenalis *was the only pathogenic parasite found in the faeces. *G. duodenalis* cysts were found in 8 out of the 316 samples examined by microscopy (2.5%), corresponding to four of the schools included in this work. Furthermore one family member (brother), from one of the infected children, was also infected with *G. duodenalis*.

### 3.2. Genotyping Characterization

**Figure 1:**
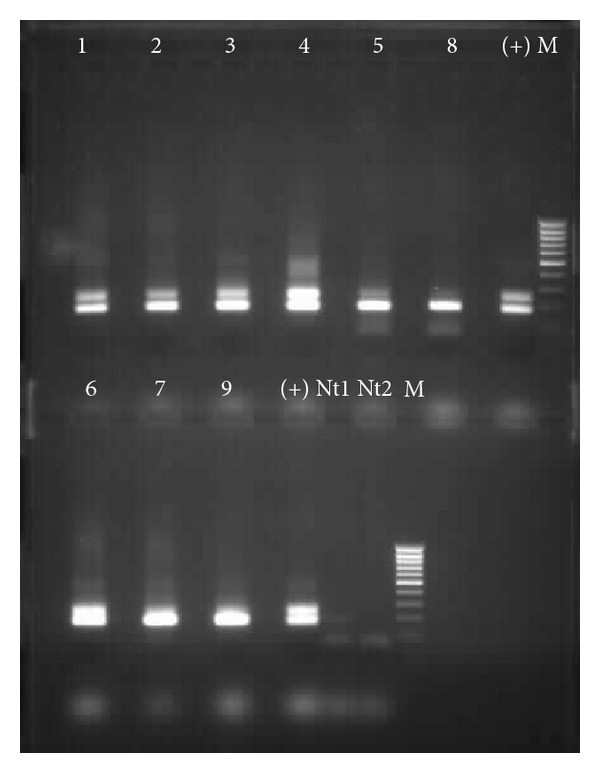
Electrophoretic separation of *ssu-rRNA* PCR products (175 bp). Lanes 1–9, *G. duodenalis* positive samples through microscopy; lanes (+), positive control (*G. duodenalis* DNA, strain Portland-1, ATCC 30888DTM LGC Promochem); lanes M, 100 bp ladder; lanes Nt1 and Nt2, negatives controls (no DNA) from the first and the second PCR reaction, respectively.

**Figure 2:**
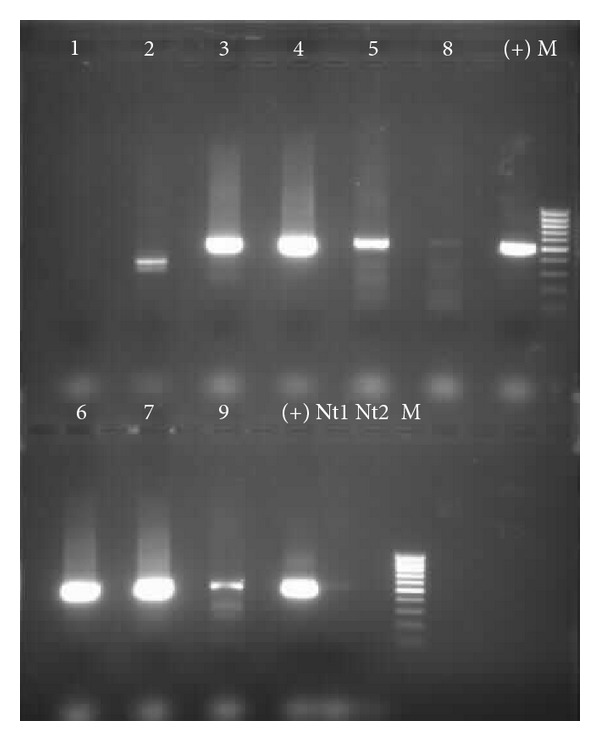
Electrophoretic separation of **β*-giardin* PCR products (511 bp). Lanes 1–9, *G. duodenalis* positive samples through microscopy; lanes (+), positive control (*G. duodenalis* DNA, strain Portland-1, ATCC 30888DTM LGC Promochem); lanes M, 100 bp ladder; lanes Nt1 and Nt2, negatives controls (no DNA) from the first and the second PCR reaction, respectively.

The nine positive samples for *G. duodenalis* were successfully amplified for *ssu-rRNA* fragment gene, and for the *bg* gene only seven samples were amplified (77.8%, 7/9) (Figures 1 and 2).

*Ssu*-*rRNA* sequences obtained in this study were compared with homologous sequences found in GenBank using BLAST. Six samples belong to assemblage A and three to B (Table 1). Sequences obtained for *bg* gene were also compared with public sequences from GenBank using BLAST. Additionally, *bg* sequences were analysed for subassemblage discrimination according to the genetic polymorphisms described elsewhere [23]. Isolates 3, 4 and 5 belong to subassemblage A2, while the other isolate (6) belongs to subassemblage A3 (Table 1).

For the remaining two isolates, 7 and 8, belonging to assemblage B, it was not possible to determine the respective subassemblage due to the high nucleotide variability observed in the chromatogram.

New stool samples were collected from all infected children after the complete treatment and analyzed. No parasite was detected by microscopy.

### 3.3. Clinical Data

At the time of the stool sample collection only four children reported symptoms (1, 3, 4, 5), including lack of appetite, abdominal pain, and flatulence (Table 1).

Case number 9, the one with intermittent diarrhea, was the only case of moderate malnutrition (BMI 12; −2 < z score < −3).

The medical examination was normal in four children (3, 6, 7, and 8). Abdominal distension and pain on deep palpation were observed in the remaining five (Table 1).

### 3.4. Genetic Assemblage and Clinical Presentation

From the three children infected with *G. duodenalis* assemblage B, two presented a normal medical examination with no symptoms (7 and 8), while the other one (1) presented abdominal distension and referred lack of appetite as a symptom. For the six children infected with assemblage A, two had a normal medical examination (3 and 6) and three referred no symptoms (2, 6, and 9). All these results are described in Table 1.

## 4. Discussion

The number of children found infected with *G. duodenalis* in this study (8/316; 2.5%) was similar to other studies conducted in Northern and Centre of Portugal where 3%, 3.7%, and 1.9%, respectively [13, 14, 17] were infected. These results are in agreement with the reported prevalence for this parasite in developed countries [12].

The use of microscopy as the only diagnostic procedure for detecting *G. duodenalis* may be considered as a limitation of this study. However, the use of three stool samples allow the detection of over 90% of infection [24], which is very similar to the sensitivities recently reported for rapid diagnostic tests [25], while one stool sample will allow the detection of 60 to 80%, and the analysis of two stool samples will allow the detection of 80 to 90% [24]. In this study at least two stool samples were obtained from all the children and three samples for more than 80% of the enrolled children. Furthermore microscopy has the additional advantage of allowing the detection of other intestinal parasites [26].

In our study assemblage A was more frequent, with six isolates, while assemblage B was detected in three children, which is the opposite pattern reported worldwide with assemblage B appearing more common [11, 12]. Subassemblage determination was only possible for assemblage A positive samples, as B samples were impossible to subtype due to the presence of double peaks at specific position in the chromatogram. The difficulty of subtyping assemblage B has been reported [18, 27]. The importance of being able to subtype is especially relevant when a source of infection must be traced or when a distinction between reinfection/new infection is mandatory for therapeutic control. For instance, in this study, two brothers were infected with the same subassemblage (A2) of *G. duodenalis*, suggesting a common source of infection.

Another interesting data obtained from this work was the finding of a child (case 9) with malnutrition (moderate), which is a common situation in developing countries [6]. This child also corresponds to the only one complaining of intermittent diarrhea. In this case chronic infection could have contributed to the child nutritional status.

While there has been a growing interest in the molecular characterization of *G. duodenalis*, there is still a lack of clear association between the assemblage and the clinical outcome, with contradictory results. A study conducted in the Netherlands found a strong correlation between assemblage A and mild, intermittent diarrhea and assemblage B with severe and persistent diarrhea [10]. Other studies conducted in Ethiopia and Saudi Arabia also suggest a correlation between the presence of symptoms and infection with the assemblage [28, 29]. On the other hand, a study performed in Australia revealed that children infected with *G. duodenalis* isolates from assemblage A were 26 times more likely to have diarrhea than children with assemblage B [20]. Supporting these results other works in Bangladesh and Spain also showed a statistical association between assemblage A and symptomatic infections and between assemblage B and asymptomatic infections [30, 31]. In what concerns to Portugal, the data obtained by Sousa et al. [15] supports that *G. duodenalis* belonging to assemblage A is strongly associated with symptomatic cases. In agreement with this, other work revealed a higher prevalence of assemblage B in asymptomatic children [13]. None of the children studied presented diarrhea at the moment of the stool sample collection. However, of the four symptomatic children at enrolment, three were infected with A2 subassemblage with more evident symptoms when compared with the other symptomatic children carrying B assemblage.

## 5. Conclusions

This study contributed with new and relevant data for the epidemiology of giardiasis in Portugal and challenged the pattern of assemblage B being more frequent than assemblage A, eventually suggesting that a different epidemiological profile is detected in developed countries. The fact that a brother from an infected child was also infected reinforced the importance of when testing a child or a family member; if positive, then the entire household should be screened. The study did not show an association between the clinical pattern observed and the assemblages. The low number of children infected with *G. duodenalis* does not justify the need for consistent request of stool parasitological analysis. However, special attention should be given to children reporting abdominal complains, considering that 4/9 of the infected children were symptomatic. 

## Figures and Tables

**Table 1:** Clinical data and *G. duodenalis* assemblages of the infected children.

Case	Age (years)	School	Present symptoms	Medical examination	Assemblages	
					*ssu *	*bg *
1	5.8	Musgueira (JI77)	Lack of appetite	Abdominal distension	B	NA
2	4.8	Horta Nova	No symptoms observed	Abdominal distension	A	NA
3	6.0	Horta Nova	Flatulence	Normal	A	A2
4*	9.5	Horta Nova	Abdominal pain, lack of appetite, and flatulence	Pain on deep palpation and abdominal distention	A	A2
5	6.3	Alto da Faia	Abdominal pain, lack of appetite	Abdominal distension	A	A2
6	6.3	Alto da Faia	No symptoms observed	Normal	A	A3
7	3.9	Ameixoeira	No symptoms observed	Normal	B	B**
8	5.8	Ameixoeira	No symptoms observed	Normal	B	B**
9	4.3	Ameixoeira	No symptoms observed	Abdominal distension	A	NI

*This children was a family member (brother) of participant 3.

**It was not possible to subtype assemblage B due to high level of polymorphism observed.

NA: not amplified.

NI: not identified. Although successfully amplified for *bg* gene, this sample did not present enough quality for sequentiation.
